# Esketamine in resistant depression: a case report

**DOI:** 10.1192/j.eurpsy.2024.1122

**Published:** 2024-08-27

**Authors:** L. Carrión Expósito, G. Chauca Chauca, R. Galan Armenteros, M. Rodriguez Lopez

**Affiliations:** ^1^Psiquiatra; ^2^Enfermera, Córdoba, Spain

## Abstract

**Introduction:**

Major depressive disorder is a common psychiatric condition affecting around 264 million people worldwide (WHO: Depression Fact Sheet. [Apr;2021]). Despite pharmacological advances, many patients still do not respond to antidepressant treatment or do so partially.

It is estimated that only 50-70% of patients respond to the initial antidepressant treatment according to the STAR-D study. 15% percent of cases do not respond significantly to various pharmacological and psychotherapeutic attempts (Rush AJ et. STAR*D report. al Am J Psychiatry). The current consensus places resistant depression for a practical approach in one that has been treated with two different antidepressant strategies in adequate doses and time and has not been remitted (Souery D et el, Treatment-resistant depression. J Clin Psychiatry 2006). We present a clinical case of a patient with Major Depressive Disorder, resistant to several therapeutic lines, in which intranasal esketamine was initiated.

**Objectives:**

The main objective is to report the result of treatment with esketamine in a clinical case.

**Methods:**

This work analyze the clinical evolution and reponse of a 62-year-old patient after initiating intranasal esketamine.

This is a patient with a single depressive episode, with no personal psychiatric history of interest that, after exhausting several options of pharmacological and non-pharmacological treatment.

Regulated psychotherapy based on cognitive behavioral therapy was carried out along with different pharmacological strategies according to the recommendations of the main clinical guidelines: antidepressant dose increase, antidepressant change, combination of several antidepressants and potentiation with another drug. We measured clinical changes with MADRS Scale (Montgomery-Asberg Depression Rating Scale) at diferent times.

**Results:**

From the fifth administration of esketamine the patient presented a clear improvement. At three months, the score on the MADRS scale improved markedly and at 6 months, the patient reported euthymia.

Score MADRS:

- Basal 46
- 3 Months 14
- 6 Months 1

As for the adverse effects, the patient presented in all administrations very mild dizziness.

**Conclusions:**

The use of esketamine is a new therapeutic approach, being fast, safe and well tolerated in patients with depression who do not respond to other treatments (Sapkota A et al. Efficacy and Safety of Intranasal Esketamine in Treatment-Resistant Depression in Adults: A Systematic Review. Cureus.2021 Aug 21;13(8)). In our patient has proven to be effective and fast.

**Disclosure of Interest:**

None Declared

